# Modulation of GABA_B_ receptors in the insula bidirectionally affects associative memory of epilectic rats in both spatial and non-spatial operant tasks

**DOI:** 10.3389/fnbeh.2022.1042227

**Published:** 2023-01-04

**Authors:** Nan Wu, Tao Sun, Xin Wu, Hongguang Chen, Zhen Zhang

**Affiliations:** ^1^Department of Neurosurgery, Tianjin Children’s Hospital (Children’s Hospital of Tianjin University), Tianjin, China; ^2^Ningxia Key Laboratory of Cerebrocranial Disease, Incubation Base of National Key Laboratory, Ningxia Medical University, Yinchuan, China; ^3^Department of Neurosurgery, The Affiliated Yantai Yuhuangding Hospital of Qingdao University, Yantai, Shandong, China

**Keywords:** GABA_B_R, insula, Intellicage, epilepsy, operant associative memory

## Abstract

**Background:**

Stimulation of gamma-aminobutyric acid (GABA) activity through GABA receptor agonists is the basic mechanism of many anticonvulsant drugs. Nevertheless, many of these GABergic drugs have adverse cognitive effects. We previously found that GABAB receptors (GABA_B_Rs) in the insula regulate operant associative memory in healthy rats. The present study aimed at investigating the effects of GABA_B_R modulation in the insula on operant associative memory in epileptic rats, along with the underlying mechanisms.

**Methods:**

The lithium-pilocarpine model of temporal lobe epilepsy (TLE) was established in male Sprague–Dawley rats. A 22-gauge stainless-steel guide cannula was surgically implanted into the granular insula cortex of the epileptic rats. Baclofen (125 ng/μl, 1 μl), CGP35348 (12.5 μg/μl, 1 μl), or saline (1 μl) was slowly infused through the guide cannula. The Intellicage automated behavioral testing system was used to evaluate operant associative memory of the epileptic rats, including non-spatial operant tasks (basic nosepoke learning and skilled nosepoke learning) and spatial operant tasks (chamber position learning). The expression of the GABA_B_R subunits GB1 and GB2 in the insula was examined by immunofluorescence and Western blotting.

**Results:**

The Intellicage tests demonstrated that baclofen significantly impaired basic nosepoke learning, skilled nosepoke learning and chamber position learning of the epileptic rats, while CGP35348 boosted these functions. Immunofluorescence staining revealed that GB1 and GB2 were expressed in the insula of the epileptic rats, and Western blotting analysis showed that baclofen enhanced while CGP35348 inhibited the expression of these subunits.

**Conclusion:**

GABA_B_Rs in the insula bidirectionally regulate both spatial and non-spatial operant associative memory of epileptic rats. Effects of GABA_B_Rs on cognition should be taken into account when evaluating new possible treatments for people with epilepsy.

## Introduction

The gamma-aminobutyric acid (GABA) is the main inhibitory neurotransmitter in the brain and a major player in the pathogenesis of epilepsy (Treiman, 2001; Sperk et al., 2004). When the balance between the inhibitory tone and neuronal excitation is perturbed, epileptic seizures may arise. GABA acts through two classes of receptors: GABA_A_Rs (ligand-gated ion channels) and GABA_B_Rs (G-protein coupled receptors). Several studies have reported the critical role of GABA_A_Rs in epileptogenesis (Chuang and Reddy, 2018; Maljevic et al., 2019), and a number of anticonvulsants, such as phenobarbital, valproic acid, benzodiazepines, and topiramate, act through GABA_A_Rs, potentiating the inhibitory effects of GABA (Jankovic et al., 2021). However, these GABA_A_R modulators can have adverse effects on the cognitive functions of patients. For example, topiramate is known to cause treatment-emergent adverse events on cognition in epileptic patients (Mula, 2012). These issues with GABA_A_R-targeting anticonvulsants have prompted the search for new GABA modulators with an improved therapeutic profile, including allosteric GABA_A_R agonists (Zeman et al., 2016; Jankovic et al., 2021) or selective GABA_B_R modulators (Avoli and Levesque, 2021).

Emerging evidence supports the involvement of GABA_B_Rs in epileptogenesis. In humans, a GABA_B_ receptor (GABA_B_R) polymorphism (G1465A) has been associated with a high risk of temporal lobe epilepsy (TLE) and disease severity in TLE patients (Gambardella et al., 2003). Moreover, GABA_B_R expression and efficacy were downregulated in human TLE cortical tissues (Teichgraber et al., 2009). In a kindling-induced rat model of epilepsy, it has been shown that stimulation of GABAergic neurotransmission through GABA_B_R agonist baclofen (BLF) had an anti-convulsant effect, while inhibition of GABAergic activity through GABA_B_R antagonist CGP35348 had a pro-convulsant effect in developing rats of 12 and 25 days (Mares and Slamberová, 2006). Furthermore, BLF showed an anti-convulsant effect in pentylenetetrazol-induced model of epilepsy in developing rats of 7, 12, 18, and 25 days (Kubová et al., 1996). In adult rats, BLF reduced pentylenetetrazol-induced seizures (Chen et al., 2004) and electroshock-induced seizures (Hyder Pottoo et al., 2022). Similarly, BLF reduced seizures in a mouse pentylenetetrazole kindling model of epilepsy (De Sarro et al., 2000).

Stimulation of GABAergic activity through GABA receptor agonists is the basic mechanism of many anticonvulsant drugs and represents a useful therapeutic strategy for people with epilepsy (Treiman, 2001; Jankovic et al., 2021).

Nevertheless, GABAergic neurotransmission also has an important role in memory processes, with, generally, agonists of GABA receptors impairing cognitive function and antagonists potentiating it (Makkar et al., 2010; Kasten and Boehm, 2015; Heaney and Kinney, 2016). Hence, treatment with the agonist BLF, especially if chronic, could lead to cognitive impairment. Indeed, in healthy rats, it has been observed that BLF impaired spatial memory (Nakagawa et al., 1995; Nakagawa and Takashima, 1997; Arolfo et al., 1998; Deng et al., 2009; Kumar et al., 2017). Additionally, our group found that BLF impaired several operant learning tasks (Wu et al., 2017).

The aim of the present study is to understand if BLF may lead to cognitive impairments also in epileptic rats. To achieve this goal, we tested the effect of BLF on the memory of lithium chloride (LiCl)-pilocarpine-induced epileptic rats, a model of temporal lobe epilepsy. In order to comprehend if the memory function of epilectic rats is bidirectionally regulated by GABAergic neurotransmission, and have a specular confirmation of the association between GABAergic neurotransmission and memory function, we also tested the cognitive performance of rats treated with the GABA receptor antagonist CGP35348 (in a non-convulsive dosage).

TLE is the most common form of focal epilepsy (Vinti et al., 2021). The insula, also known as the “hidden fifth lobe,” is a part of the cerebral cortex positioned deep within the lateral fissure. The insular lobe has a relevant role in TLE, with epileptic activity often invading it from the temporal cortex and in some cases even originating in it (Isnard et al., 2000; Blauwblomme et al., 2013; Barba et al., 2017). Regarding its functions, the insula has long been associated with taste memory (Yiannakas and Rosenblum, 2017) and has been recently linked to non-gustatory learning, in particular object recognition memory formation (Martin et al., 2012, 2021; Korczyn et al., 2013; Bermudez-Rattoni, 2014; Titiz et al., 2014). Additionally, pharmacological inhibition of insula in mice impaired associative memory, disrupting conditioned responses to reward-associated cues, in particular cue-triggered reward approach (Kusumoto-Yoshida et al., 2015). Associative memory can be studied in animal models through two different conditioning paradigms. While classical (Pavlovian) conditioning features the formation of an association between two stimuli (an S-S association), operant conditioning features an association between a stimulus and a behavioral response (an S-R association) (d’Isa et al., 2011). Our group previously found that the insula is involved in operant associative learning of conditioned nosepoking via GABA_B_Rs (Wu et al., 2017). In particular, we observed that intra-insula infusion of the GABA_B_R agonist baclofen impaired position learning, punitive learning, and punitive reversal learning in normal rats, while the antagonist CGP35348 enhanced these learning abilities (Wu et al., 2017), indicating that proper functioning of GABA_B_Rs in the insula is critical for maintaining operant associative memory. However, what effect the modulation of GABA_B_Rs in the insula of epileptic rats could have on memory function is yet to be investigated.

In the present study, we used the Intellicage system to assess the effects of intra-insula infusion of baclofen or CGP35348 on operant associative memory functions in LiCl-pilocarpine-induced epileptic rats. Also, the underlying insular GABA_B_R expression levels were analyzed. Our current findings shed new light on the cognitive effects of GABAergic drugs in epilepsy, indicating a memory-impairing effect for GABA_B_R agonist baclofen and a cognitive enhancing effect for GABA_B_R antagonist CGP35348.

## Materials and methods

### Reagents

Primary antibodies against GABA_B_R subunits GB_1_ and GB_2_ were purchased from Abcam (UK). The selective GABA_B_R agonist baclofen and antagonist CGP35348 were obtained from Sigma–Aldrich (USA).

### Animals

Male Sprague–Dawley rats (6–8-week-old, 250–300 g)(10 rats/group) were obtained from the Animal Center of Ningxia Medical University (Yinchuan, Ningxia, China). Rats were kept under a 12:12 h light/dark cycle (lights on at 8 a.m.) with free access to food and water. Each rat was housed in a separate cage in order to avoid damage to the cannula implants and harm to the rats. All animal studies were conducted in accordance with the Regulations of Experimental Animal Administration issued by the State Committee of Science and Technology of China on October 31, 1988. All animal protocols were approved by the Ethics Committee of the Animal Center of Ningxia Medical University.

### TLE model

A lithium-pilocarpine TLE model was established as described previously (Andre et al., 2001). Briefly, lithium chloride (LiCl) in saline (127 mg/kg) was injected into the rat abdominal cavity. After 18 h, hyoscyamine sulfate [1 mg/kg, intraperitoneally (i.p.)] was administered to mitigate the peripheral effects of pilocarpine, and 30 min later, pilocarpine (30 mg/kg, i.p.) was injected to induce status epilepticus (SE). If the rat did not exhibit behavioral seizures (≥class 4 on the scale of Racine) within 30 min of pilocarpine injection, an additional dose (10 mg/kg, i.p.) was administered every 30 min until clinical signs were observed. The total amount of pilocarpine administered in each rat did not exceed 60 mg/kg, while diazepam (10 mg/kg, i.p.) was administered to stop the seizure 1 h after the onset of SE or after the rat exhibited dehydration symptoms. During the induction session, each rat was scored for epileptic signs according to Racine’s scale. Successful induction was defined as induction of epileptic seizures ≥class 4 of the Racine scale. With the described protocol, we obtained a success rate of over 70%. For the present work, a pool of over 75 rats underwent induction. Among the rats showing successful induction, 50 rats were randomly selected as experimental animals. No rat died during induction nor during the whole period of experimental testing.

### Surgery

After 1 week of acclimatization, the epileptic rats were randomly divided into five groups (10 rats/group): control, sham, saline (NaCl), baclofen (BLF), and CGP35348 (CGP). The rats in the sham, NaCl, BLF, and CGP groups underwent surgical implantation of a bilateral cannula aimed at the granular insular cortex according to a standardized protocol (Balderas et al., 2015). Briefly, the animals were anesthetized with 10% chloral hydrate (4 ml/kg, i.p.) and mounted on a stereotaxic frame. A 22-gauge stainless-steel guide cannula was inserted into the granular insula cortex according to coordinates from the Paxinos and Watson brain atlas (mm from bregma: AP = + 1.2; ML = ± 5.5; mm from skull surface: DV = −6.5) (Paxinos and Watson, 1986). The cannula was anchored to the skull using stainless steel screws and acrylic cement.

### Bilateral intra-insula microinjection

The animals were allowed to recover for 14 days after the cannula implantation surgery. In order to evaluate the effects on memory function, the rats received slow bilateral intra-insula microinjection (1 μl/0.5 min) as follows: sham, no treatment; NaCl, NaCl at 0.3 nmol/μl; BLF, baclofen at 125 ng/μl (St Onge and Floresco, 2010); CGP, CGP35348 at 12.5 μg/μl (Ataie et al., 2013). The injection needle (50 G) was left in place for 1 min post-injection to prevent backflow. Infusions were administered on each day of behavioral testing. After the infusion was complete, the rats were allowed to rest for 30 min before behavioral testing.

### Signal transponder implantation

Signal transponders were implanted above the scapula using the injector system of the Intellicage device. The implantation was carried out under 10% chloral hydrate (4 ml/kg, i.p.) 14 days after the surgery and 24 h prior to introduction into the Intellicage. The transponders were used to follow the movement of the rats during behavioral testing (Urbach et al., 2014). Visits of each rat to each corner of the Intellicage were detected through radio-frequency identification (RFID) antennas installed in the cage.

### Intellicage

The Intellicage (TSE Systems GmbH, Germany) is a new automated group-testing system that allows standardized rodent behavioral phenotyping in a social context and without interaction with the experimenter during the test. It features four operant conditioning chambers positioned in each corner of the cage. Each chamber is equipped with a transponder-reader antenna that registers the number of visits by the rat. Inside each chamber there are two doors (the left and the right door), each giving access to a water-drinking bottle. Each water bottle is separately gated by one door, and door opening is controlled by an infrared beam-break sensor that detects correct nosepoking (inserting the nose in a small hole near the door). The number of chamber entries and nosepokes are automatically recorded and processed with the Intellicage software. Total dimensions of the rat Intellicage are: 118 cm x 118 cm x 46 cm (rat system, center cage + corner + water bottles). Currently, the Intellicage system is one of the most advanced commercial apparatuses for automated rodent behavioral testing (Kiryk et al., 2020; Iman et al., 2021), and its first application in behavioral research on insular functions was published by our research group (Wu et al., 2017).

### Behavioral test

The rats were transferred to the Intellicage 2 weeks post-surgery. The behavioral information was collected from 9:00 to 12:00 a.m. Each rat was tested in daily sessions of 30 min. Rats were tested in groups of 10, randomized by experimental group (2 for each of the 5 experimental groups). Each day, 5 sessions were performed, with 10 rats in each session (50 total rats tested each day). The Intellicage was cleaned with ethanol 70% at the end of each session. Each rat was tested in one single session per day. The rats were removed from the Intellicage at the end of each daily testing session and maintained in their home-cage with free access to food and water until the next testing session. Water bottles were removed from the home-cages from 7:00 to 9:00 a.m. of each testing day. Although absence of water for 2 h does not cause an actual physiological water deprivation in rats, the disappearance of the familiar water bottles from the home-cage shortly before the test was aimed at promoting research of alternative sources of water in the Intellicage.

**FIGURE 1:**
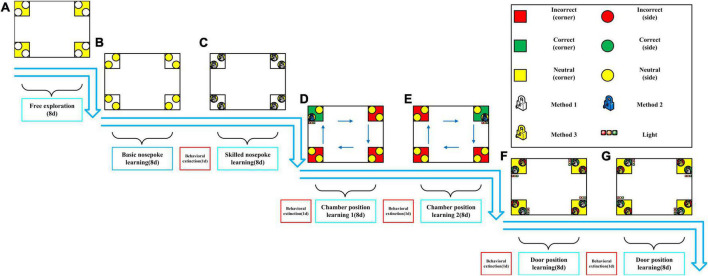
Intellicage behavioral testing experimental design. **(A)** Free exploration. **(B)** Basic nosepoke learning. **(C)** Skilled nosepoke learning. **(D)** Chamber position learning 1. **(E)** Chamber position learning 2. **(F)** Door position learning 1. **(G)** Door position learning 2.

Intellicage testing experimental design (Figure 1):

1. Free exploration: 8 days in the Intellicage with free access to all water bottles (all doors open). The number of chamber visits was recorded to evaluate exploratory behavior of the animals. This phase also served as familiarization with the Intellicage environment and with the other rats. The subsequent learning tasks (nosepoke learning, chamber position learning and door position learning) were performed only after these 8-days of contextual and social familiarization.
2. Basic nosepoke learning: 8 days in the Intellicage with access to water bottles granted by one correct nosepoke. In each chamber, doors were closed and could be opened only by nosepoking. Number of nosepokes was recorded.
3. Behavioral extinction: 1 day in the Intellicage with free access to all water bottles (all doors open) to extinguish the previous learning.
4. Skilled nosepoke learning: 8 days in the Intellicage with access to water bottles granted by five correct nosepokes. In each chamber, doors were closed and could be opened only by nosepoking. Number of nosepokes was recorded.
5. Behavioral extinction: Same as in step 3.
6. Chamber position learning 1: A chamber is located in each of the four corners of the Intellicage. The least visited chamber identified from the skilled nosepoke learning test was designated as “correct,” and the three remaining chambers were designated as “incorrect.” In order to avoid any possible spatial bias, each rat was tested in all four possible spatial configurations (“correct” corner in North-West, North-East, South-East or South-West). Four consecutive 2-day tests were performed. The “correct” chamber was rotated 90°clockwise every 2 days. The rats had access to all chambers, but drinking water was allowed only in the “correct” chamber. In each chamber, the left door was open, while the right door was closed. Rats could obtain water only through the left bottle of the “correct” chamber (the left bottles of the three “incorrect” chambers were empty). Three multi-color LEDs over the left door of the “correct” chamber served as visual cues for the correct site.

The number of visits to each chamber was recorded. The experiment was completed in 8 days. Number of visits and percentage of visits to the “correct” chamber were used to evaluate chamber position learning.

1. Behavioral extinction: Same as in step 3.
2. Chamber position learning 2: The testing conditions were similar to those in chamber position learning 1, except that the rats could access the water bottle only through the right door of the “correct” chamber. In each chamber, right doors were open and left doors were closed. Three multi-color LEDs over the right door of the “correct” chamber served as visual cues for the correct site.
3. Behavioral extinction: Same as in step 3.
4. Door position learning 1: The left side of each chamber was designated as the “correct” side and the right side as the “incorrect” side. The door of the “correct” side would open after five nosepokes and stay open for 10 s, and door at the “incorrect” side would also open after five nosepokes but stay open for only 3 s. Three multi-color LEDs over the left doors served as visual cues for the correct sites. Water drinking was allowed at both sites in all chambers. Number of nosepokes to each door was recorded. The experiment was completed in 8 days.
5. Behavioral extinction: Same as in step 3.
6. Door position learning 2: The testing conditions were the same as those in door position learning 1, except that the right side of each chamber was designated as the “correct” side and the left side as the “incorrect” side. Three multi-color LEDs over the right doors served as visual cues for the correct sites.

### Tissue sample collection

After completing all behavioral tests in Intellicage, on the day after the rats were euthanized under 10% chloral hydrate. Left and right insula tissues were collected for immunofluorescence staining and Western blot analysis.

### Immunofluorescence staining

The insular tissues were fixed in 4% paraformaldehyde, dehydrated, embedded in optimal cutting temperature (OCT) compound, and sectioned. GB1 and GB2 were detected by immunofluorescence staining. Briefly, after antigen retrieval in citric acid buffer, the sections were blocked in serum and incubated with anti-GB1 (1:300) or anti-GB2 (1:500) antibody at 4°C overnight. After washing in phosphate-buffered saline (PBS), the samples were incubated at room temperature with a FITC-labeled secondary antibody for 1 h. The unbound antibody was removed with PBS washes. After blocking with an anti-quencher, the samples were analyzed by immunofluorescence imaging. The cell nuclei were counterstained with DAPI. The imaging data were processed with the ImageJ 1.48 analysis system.

### Western blotting

The insula tissues were placed on ice and lysed in lysis buffer. The total protein content was determined using the BCA method. The proteins were separated by SDS/PAGE and transferred to PVDF membranes. After blocking with 5% non-fat milk for 1 h, the membranes were probed with anti-GB1 (1:300) or anti-GB2 (1:500) antibody at 4°C overnight and incubated with an IRDye 800CW dye-labeled secondary antibody (1:5,000). The immunoreactive bands were detected on an Odyssey infrared laser imaging system and quantified by gray intensity analysis. The protein levels were normalized to those of GADPH.

### Statistical analysis

All data are presented as the mean ± standard error of mean (SEM) and analyzed using the SPSS 21.0 software. Overall behavioral responses (total responses throughout all days of testing) and Western blotting results were analyzed using one-way analysis of variance (ANOVA), followed by Fisher’s Least Significant Difference (LSD) *post hoc* test in case of significant effect of experimental group. Daily behavioral responses were analyzed through a two way ANOVA for repeated measures, followed by Fisher’s LSD *post hoc* test in case of significant effect of experimental group. For percentages of correct visits, chance level analysis was performed by comparing the percentages of each experimental group against chance level (25%) through a one-sample *t*-test. In all analyses, a *p*-value < 0.05 was considered statistically significant.

## Results

### Intra-insula baclofen impaired while CGP35348 boosted the operant associative memory of TLE rats

**FIGURE 2:**
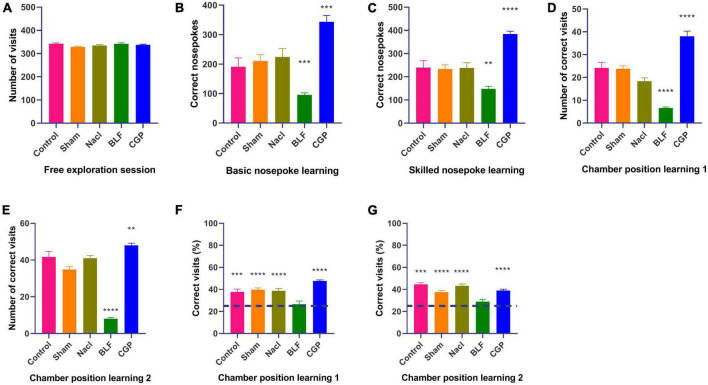
Intellicage behavioral testing: overall results (sum of all 8 days of testing). **(A)** Number of visits in free exploration. **(B)** Number of nosepokes in basic nosepoke learning. **(C)** Number of nosepokes in skilled nosepoke learning. **(D)** Number of correct visits in chamber position learning 1. **(E)** Number of correct visits in chamber position learning 2. **(F)** Percentage of correct visits in chamber position learning 1. **(G)** Percentage of correct visits in chamber position learning 2. *N* = 10 rats/group. The asterisks indicate significance against the NaCl group in **(A–E)** or against chance level (25%) in **(F,G)** **p = 0.01; ***p = 0.001; ****p = 0.0001.

Rats underwent a series of behavioral protocols in the Intellicage as described in Figure 1: a free exploration test, two non-spatial operant tasks (basic nosepoke learning and skilled nosepoke learning), two spatial operant tasks (chamber position learning 1 and chamber position learning 2) and finally two tasks in which both nosepoking and spatial discrimination were required (door position learning 1 and door position learning 2). Overall behavioral responses totalized over the course of all days of testing are showed in Figure 2.

During the 8-day free exploration test, in which all doors were open, the five groups of epileptic rats showed similar exploratory capacities under unrestricted conditions, as evaluated by the number of visits to the water bottle chambers (*F*(4,45) = 2.006, *p* = 0.110; Figure 2A). However, during the basic nosepoke learning test, when the rats had to learn to perform a correct response (a nosepoke) to open a door to access the water bottle, a significant effect of experimental group was found (*F*(4,45) = 14.708, *p* < 0.0001; Figure 2B). The BLF rats nosepoked significantly less (*p* = 0.0003), while the CGP rats nosepoked significantly more (*p* = 0.0007) than the NaCl rats, indicating that baclofen decreased while CGP35348 increased the basic nosepoke learning ability of epileptic rats. Similarly, a significant effect of experimental group was found also in skilled nosepoke learning (*F*(4,45) = 17.919, *p* < 0.0001; Figure 2C), in which rats had to nosepoke five times to access the water bottles. BLF rats exhibited a lower (*p* = 0.003) while the CGP rats displayed a higher (*p* < 0.0001) number of nosepokes than the NaCl rats.

Subsequenly, we tested the rats in two spatial tasks: chamber position learning 1 and chamber position learning 2 (Figures 2D, E), in which a bottle with water was placed in one of the four corner chambers (the “correct chamber”), while the other three corner chambers contained empty bottles (the “incorrect” chambers). Rats had to learn the position of the bottle with water and simply visit the “correct” chamber (no nosepoking required) to drink. Effect of experimental group was significant for both spatial tasks (chamber position learning 1: *F*(4,45) = 43.741, *p* < 0.0001; chamber position learning 2: *F*(4,45) = 79.111, *p* < 0.0001). In both spatial tasks, baclofen impaired the performance, while CGP35348 improved it. In comparison with the NaCl group, BLF rats showed a significant decrease in the total number of correct visits (chamber position learning 1: *p* < 0.0001; chamber position learning 2: *p* < 0.0001), while CGP rats displayed a significant increase (chamber position learning 1: *p* < 0.0001; chamber position learning 2: *p* = 0.007).

Additionally, in order to perform a chance level analysis, we proportioned the number of visits in the correct corner to the total number of visits (number correct visits + number of incorrect visits), obtaining the percentage of visits to the correct corner (Figures 2F, G). Since, in the spatial tasks, one corner was correct and 3 corners were incorrect, for the percentage of correct visits the chance level performance was 25%. Compared against chance level, all groups apart from the BLF rats showed significant learning, in both chamber position learning 1 (control: *p* = 0.0008; sham: *p* < 0.0001; NaCl: *p* < 0.0001; BLF: *p* = 0.564; CGP: *p* < 0.0001) and chamber position learning 2 (control: *p* = 0.0008; sham: *p* < 0.0001; NaCl: *p* < 0.0001; BLF: *p* = 0.074; CGP: *p* < 0.0001). Moreover, in comparison with percentages of NaCl rats, percentages of BLF were significantly lower in chamber position learning 1 (*p* = 0.0002) and chamber position learning 2 (*p* < 0.0001), while percentages of CGP were significantly higher in chamber position learning 1 (*p* = 0.004).

Regarding door position learning, the experiments were not valid as the control groups developed a significant preference for the wrong side. Hence it was not possible to use this test to evaluate the memory of the experimental groups. Since the experiments are invalid, we are not showing the results. Probably the experimental protocol did not work because the difference between the value of the reward of the two sides (3 s of water vs 10 s of water) was insufficient to induce a preference for the correct side in the rats of the control groups. We further comment on this issue in the Discussion.

**FIGURE 3:**
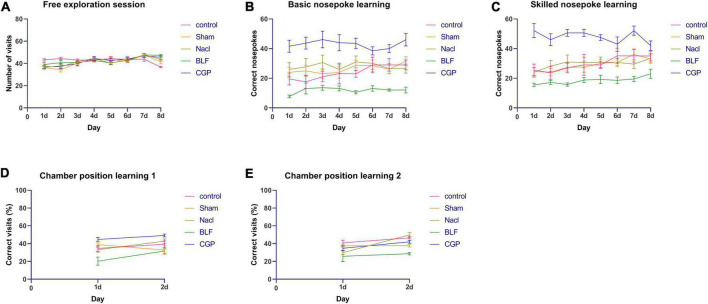
Intellicage behavioral testing: daily results. **(A)** Number of visits in free exploration. **(B)** Number of nosepokes in basic nosepoke learning. **(C)** Number of nosepokes in skilled nosepoke learning. **(D)** Percentage of correct visits in chamber position learning 1. **(E)** Percentage of correct visits in chamber position learning 2. *N* = 10 rats/group. *N* = 10 rats/group.

Additionally, we performed a single day analysis in order to investigate in more detail differences between experimental groups. For the free exploration paradigm (Figure 3A), two-way ANOVA for repeated measures confirmed no significant effect of experimental group (*F*(4,45) = 2.006; *p* = 0.110).

For basic nosepoke learning (Figure 3B), a significant effect of experimental group was found (*F*(4,45) = 14.708; *p* < 0.0001). Compared with NaCl, BLF rats showed a significantly lower performance on day 1 (*p* = 0.0009), day 2 (*p* = 0.025), day 3 (*p* = 0.005), day 5 (*p* < 0.0001), day 6 (*p* = 0.003), day 7 (*p* = 0.002) and day 8 (*p* = 0.003), whereas CGP rats showed a significantly higher performance on day 1 (*p* = 0.004), day 2 (*p* = 0.013), day 3 (*p* = 0.10), day 4 (*p* = 0.008), day 5 (*p* = 0.006), day 7 (*p* = 0.003) and day 8 (*p* < 0.0001). Performance of CGP rats was significantly higher than the one of BLF rats on day 1 (*p* < 0.0001), day 2 (*p* < 0.0001), day 3 (*p* < 0.0001), day 4 (*p* < 0.0001), day 5 (*p* < 0.0001), day 6 (*p* < 0.0001), day 7 (*p* < 0.0001) and day 8 (*p* < 0.0001).

For skilled nosepoke learning (Figure 3C), a significant effect of experimental group was found (*F*(4,45) = 17.919; *p* < 0.0001). Compared with NaCl, BLF rats showed a significantly lower performance on day 2 (*p* = 0.026), day 3 (*p* = 0.001), day 4 (*p* = 0.047), day 5 (*p* = 0.005), day 6 (*p* = 0.027), day 7 (*p* = 0.035) and day 8 (*p* = 0.022), whereas CGP rats showed a significantly higher performance on day 1 (*p* < 0.0001), day 2 (*p* = 0.0006), day 3 (*p* < 0.0001), day 4 (*p* = 0.001), day 5 (*p* < 0.0001) day 6 (*p* = 0.024) and day 7 (*p* < 0.0001). Performance of CGP rats was significantly higher than the one of BLF rats on day 1 (*p* < 0.0001), day 2 (*p* < 0.0001), day 3 (*p* < 0.0001), day 4 (*p* < 0.0001), day 5 (*p* < 0.0001), day 6 (*p* < 0.0001), day 7 (*p* < 0.0001) and day 8 (*p* = 0.0001).

For the spatial tasks, four 2-day tests were performed, one for each spatial configuration (correct corner in North-West, North-East, South-East or South-West), in order to avoid any possible spatial bias. The average performance for all four spatial configurations was considered for analysis. For chamber position learning 1 (Figure 3D), a significant effect of experimental group was found (*F*(4,45) = 13.505; *p* < 0.0001). Compared with NaCl, BLF rats exhibited a significantly reduced performance on day 1 (*p* = 0.005) and day 2 (*p* = 0.004), while CGP rats showed a significantly increased performance on day 1 (*p* = 0.013). Performance of CGP rats was significantly higher than the one of BLF rats on day 1 (*p* < 0.0001) and day 2 (*p* < 0.0001). For chamber position learning 2 (Figure 3E), a significant effect of experimental group was found (*F*(4,45) = 8.887; *p* < 0.0001). Compared with NaCl, on day 2, the performance of BLF rats was significantly reduced (*p* < 0.0001), while the performance of CGP rats was significantly augmented (*p* = 0.003). Performance of CGP rats was significantly higher than the one of BLF rats on day 2 (*p* < 0.0001).

Collectively, these results demonstrated that bilateral intra-insula infusion of baclofen impaired associative memory of TLE rats, while the infusion of CGP35348 boosted this function.

### GABA_B_R was expressed in the insula of TLC rats

**FIGURE 4:**
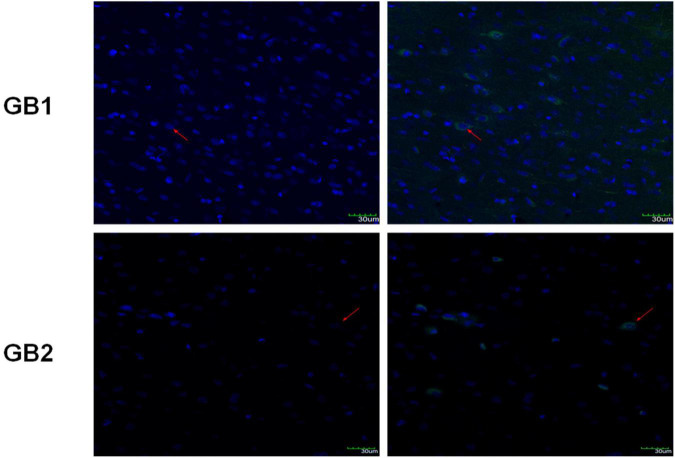
Immunofluorescence staining of insula tissues for GB1 and GB2 expression. The red arrows indicate cells showing positive staining for both GB1 and DAPI or GB2 and DAPI. Scale bars: 30 μm.

In a previous study (Wu et al., 2017), we detected GB1 and GB2, the two subunits of GABA_B_R, in the insula of normal Sprague-Dawley rats. In the present study, the immunofluorescence results revealed positive GB1 and GB2 staining in the insula tissues of the epileptic Sprague-Dawley rats (Figure 4), indicating that GABA_B_R was expressed in the insula of these rats.

### Baclofen increased while CGP35348 decreased insular GABA_B_R expression in TLC rats

**FIGURE 5:**
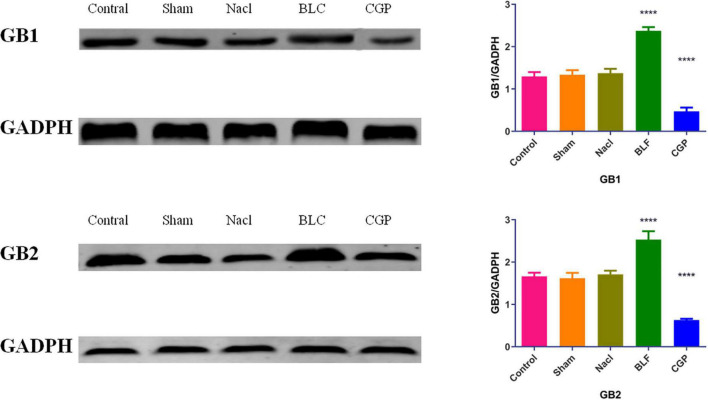
Western blot analysis of GB1 and GB2 levels in insula tissues. *N* = 10/group. The asterisks indicate significance against the NaCl group: ****p < 0.0001.

In a previous study (Wu et al., 2017), we found that baclofen increased while CGP35348 decreased GB1 and GB2 expression in the insula of normal Sprague-Dawley rats. In the present study, the expression of GB1 and GB2 was evaluated in the insula of epileptic Sprague-Dawley rats by Western blot analysis (Figure 5). A significant effect of experimental group was found for both GB1 expression (*F*(4,45) = 46.034, *p* < 0.0001) and GB2 expression (*F*(4,45) = 31.841, *p* < 0.0001). Compared to NaCl-treated rats, the baclofen-treated rats showed higher insular expression of GB1 (*p* < 0.0001) and GB2 (*p* < 0.0001), while the CGP35348-treated rats exhibited lower insular expression of GB1 (*p* < 0.0001) and GB2 (*p* < 0.0001). These findings indicate that, similarly to the effects observed in normal rats, baclofen induced while CGP35348 inhibited GABA_B_R expression in the insula of epileptic rats.

## Discussion

Many antiepileptic drugs have adverse cognitive effects, which can significantly impact the quality of life of people with epilepsy (Hermann et al., 2010). Thus, understanding the neural network involved in epilepsy treatment-associated cognitive dysfunction is critical for improved disease management. In this study, we investigated the effects of GABA_B_R modulation in the insula on the operant associative memory functions of epileptic rats. The memory functions were evaluated using the Intellicage system, one of the most advanced automated devices for rodent behavioral testing. The current experiments demonstrated that bilateral intra-insula infusion of the GABA_B_R agonist baclofen impaired operant associative memory of epileptic rats, while the infusion of the GABABR antagonist CGP35348 boosted this function. Next, we confirmed GABA_B_R expression in the insula by immunofluorescence staining. We also found that baclofen induced while CGP35348 inhibited GABA_B_R expression in the insula, further supporting the theory that the effects of baclofen and CGP35348 were mediated through GABA_B_R modulation in the insula.

Our current findings indicate that GABA_B_R modulation in the insula has a strong effect on associative memory of TLE rats, which should be taken into account when considering GABA_B_R modulators for potential epilepsy treatment.

In Intellicage experiments, two non-spatial tasks (basic nosepoke learning and skilled nosepoke learning) and two spatial tasks (chamber learning 1 and chamber learning 2) were found altered by modulation of gabaergic neurotransmission in insula. In door position learning, we were unable to obtain valid results as the control groups developed a significant preference for the wrong side, so it was not possible to use this test to evaluate the drug-treated rats. The reason for which we could not obtain valid results with this experimental protocol could be that the difference between the rewarding values of the rewards provided in the two sides (3 s of water vs 10 s of water) was insufficient to induce a preference for the correct side in the rats of the control groups. In future experiments, rather than providing as reward water in both sides, the difference between reward values of the two sides could be augmented by dispensing in one side water and in the other side a saccharin solution, which rats naturally prefer over water.

Diving deeper into cognitive processes, why does modulation of insular gabaergic neurotransmission bidirectionally alter operant learning? In which specific psychological process is insula involved? Four hypotheses can be made to explain the changes in operant learning: a) alteration of place recognition; b) alteration of cue recognition; c) alteration of the stimulus-response association; d) alteration of the rewarding value of water. All four cases may lead to an alteration of operant conditioning. The last hypothesis (d) can be discarded, since in the free exploration paradigm, in which no nosepokes were required to access the water bottles, visits to the bottles were comparable across groups. This indicates that there were no differences in locomotor activity, motivation to explore and reward value of water. The other three hypotheses remain valid possibilities. Additionally, it should be considered that multiple alterations could be present together. Both spatial operant tasks (chamber position learning 1 and 2) were altered. In these tasks, the correct sites for the behavioral response were determined by the spatial position and by the presence of spatially-specific visual cues (the three multicolor LEDs). Nevertheless, the fact that alterations were present also in the non-spatial operant tasks (basic nosepoke learning and skilled nosepoke learning), in which nosepoking (inserting the nose in a hole) is required to access water but regardless of the spatial position (any chamber and any door lead to the reward), suggests that an alteration of spatial memory alone cannot be responsible for the observed behavioral phenotype and that also an alteration of the basic ability to form stimulus-response links is present. In operant conditioning a behavioral response (as nosepoking or approach) is linked to a stimulus (as a luminous visual cue or a specific place). Insula could be involved in behavioral reactivity to the stimulus. Indeed, a previous study on mice found that insula inhibition impaired cue-reactivity (Kusumoto-Yoshida et al., 2015). Future experiments, employing specifically designed behavioral protocols, could help to dissect the role of insula in modulating these single components of operant learning.

The majority of the studies on the effects of baclofen and CGP35348 have focused on the hippocampus as the central node of memory regulation (Arolfo et al., 1998; Deng et al., 2009; Gillani et al., 2014; Lee et al., 2016). Our previous study, for the first time, showed that GABA_B_Rs in the insula are involved in memory regulation (Wu et al., 2017). The present study showed that GABA_B_Rs in the insula are also involved in the regulation of operant associative memory in epileptic rats. These findings shed light on the site and mechanisms of memory regulation and spur the development of novel treatments for patients with cognitive impairment. Importantly, in the present work we did not employ healthy rats. We focused on epilectic rats because our main aim was to understand if positive and negative modulation of insular gabaergic neurotransmission can lead to, respectively, reduced and increased memory also in epileptic rats, similarly to what we had previously found in healthy rats (Wu et al., 2017). In future experiments, it will be useful to test together six groups of rats: healthy-sham, healthy-BLF, healthy-CGP, epileptic-sham, epileptic-BLF and epileptic-CGP. The comparison between the two sham groups (receiving no drugs) will indicate if a cognitive impairment is present in epileptic rats for these tasks. On the other hand, the other groups will show if the drug-induced increase and decrease of cognitive function is of comparable size between healthy and epileptic rats. Next, we plan to investigate the role of the insula in the regulation of cognitive behavior, the underlying molecular mechanisms, and its interactions with the other brain regions of the memory network. Also, the Intellicage system would be utilized in future studies because, unlike many conventional behavioral tests that require a high degree of animal handling and interaction with the experimenter, the automated Intellicage system provides an environment that closely resembles a natural social context with minimal human interference.

In summary, we found that GABA_B_Rs in the insula bidirectionally regulate the operant associative memory of epileptic rats. Cognitive impairment induced by stimulation of GABA_B_Rs and cognitive enhancement induced by inhibition of GABA_B_Rs should be taken into account when evaluating new possible treatments for people with epilepsy.

## Data availability statement

The original contributions presented in this study are included in the article/supplementary material, further inquiries can be directed to the corresponding author.

## Ethics statement

This animal study was reviewed and approved by Rats were provided from the Animal Center of Ningxia Medical University (China) and all experiments were in accordance with the Regulations of Experimental Animal Administration issued by the State Committee of Science and Technology of China on October 31, 1988, No. (2016-124). Written informed consent was obtained from the owners for the participation of their animals in this study.

## Author contributions

NW contributed to experimental designing, planning of the experiments, performing the experiments, data analysis, and writing the manuscript. TS contributed to experimental designing, planning of the experiments, and manuscript writing. XW took part in experimental designing, planning of the experiments, data analysis, and writing the manuscript. HC contributed to experimental designing and planning of the experiments. ZZ contributed to experimental designing, planning of the experiments, data analysis, data visualization, manuscript writing, manuscript revision, and funding acquisition. All authors read and approved the final manuscript.
